# The Effects and Safety of Chinese Herbal Medicine on Blood Lipid Profiles in Placebo-Controlled Weight-Loss Trials: A Systematic Review and Meta-Analysis

**DOI:** 10.1155/2022/1368576

**Published:** 2022-01-17

**Authors:** Ann Rann Wong, Angela Wei Hong Yang, Mingdi Li, Andrew Hung, Harsharn Gill, George Binh Lenon

**Affiliations:** ^1^School of Health and Biomedical Sciences, RMIT University, Bundoora, Melbourne, Victoria, Australia; ^2^School of Science, RMIT University, Melbourne, Victoria, Australia

## Abstract

This study was conducted to assess the effects and safety of Chinese herbal medicine (CHM) on blood lipids among adults with overweight or obesity. Fourteen bibliographic databases were comprehensively searched, from their respective inceptions up to April 2021, for randomised placebo-controlled weight-loss trials using CHM formulation on total cholesterol, triglycerides, LDL cholesterol, and HDL cholesterol over ≥4 weeks. Data collection, risk of bias assessment, and statistical analyses were guided by the Cochrane Handbook (v6.1). Continuous outcomes were expressed as the mean difference with 95% confidence intervals, and categorical outcomes were expressed as a risk ratio with 95% confidence intervals. All analyses were two-tailed with a statistical significance of *p* < 0.05. Fifteen eligible studies with 1,533 participants were included in this meta-analysis. Findings from meta-analyses indicated that CHM interventions, compared to placebo, reduced triglyceride (MD −0.21 mmol/L, 95% CI −0.41 to −0.02, *I*^2^ = 81%) and increased HDL cholesterol (MD 0.16 mmol/L, 95% CI 0.04 to 0.27, *I*^2^ = 94%) over a median of 12 weeks. The reduction in total cholesterol and LDL cholesterol were not statistically significant. Furthermore, the tendency of reduced triglycerides was identified among overweight participants with high baseline triglycerides. Attrition rates and frequency of adverse events were indifferent between the two groups. CHM may provide lipid-modulating benefits on triglycerides and HDL cholesterol among participants with overweight/obesity, with the tendency for significant triglyceride reduction observed among overweight participants with high baseline triglycerides. However, rigorously conducted randomised controlled trials with larger sample sizes are required to validate these findings.

## 1. Introduction

Obesity is a chronic metabolic disease that presents a risk factor for the development of cardiovascular and metabolic complications [1, 2]. These complications are mediated, in part, by the presence of dyslipidaemia, which accounts for approximately 60–70% of the population with obesity [3]. Dyslipidaemia is characterised by abnormalities of lipid profiles including elevated levels of total cholesterol (≥200 mg/dL), triglycerides (≥150 mg/dL), and low-density lipoprotein (LDL) cholesterol (≥100 mg/dL), with low levels of high-density lipoprotein (HDL) cholesterol (<50 mg/dL for male and <40 mg/dL for female) [4]. Clinical management of dyslipidaemia ranging from lifestyle interventions to conventional medications has been proposed [5]. However, lifestyle modification as the first-line of management has faced various challenges, such as suboptimal responses and lack of adherence; thus, patients require additional drug interventions [5]. Although lipid-lowering medications confer therapeutic benefits, there has been a concern that a subgroup of patients with statin intolerance may experience muscular, cognitive, and metabolic events [6]. Therefore, many sufferers resort to natural medicines and nutraceutical compounds for managing obesity and dyslipidaemia.

Chinese herbal medicines (CHM) commonly prescribed for obesity have been reported to modulate blood lipid profiles in *in vitro* studies [7, 8]. Moreover, meta-analyses have indicated various improvements in total cholesterol, triglycerides, LDL cholesterol, and HDL cholesterol after administrating CHM formulations [9, 10] and single herbal extracts [11–13] compared to placebo, lifestyle intervention, and lipid-lowering medications. However, these studies were mainly performed on the general population with dyslipidaemia. There was limited evidence of changes in blood lipid profile among the population with obesity. Yet subjects with obesity and dyslipidaemia often have a heightened risk for developing type 2 diabetes, hypertension, and coronary heart disease [3, 14]. With the surge of obesity incidence in recent decades, more clinical research targeted to assist in the effective management of obesity and its related comorbidities are needed.

Despite various evidence supporting the clinical efficacy of herbal medicines on blood lipids, their safety and tolerability remain debatable. Several triglyceride-lowering compounds have been associated with hepatotoxicity and nephrotoxicity in experimental studies [15]. Furthermore, the underreporting of critical information on adverse events in herbal medicine randomised controlled trials (RCTs) has hampered the systematic synthesis and interpretation of herb safety profiles [16, 17]. Hence, this study aimed to evaluate the efficacy and safety of CHM on blood lipid profiles in the population with overweight or obesity by conducting a systematic review of RCTs.

## 2. Materials and Methods

This review was guided by the Cochrane Handbook for Systematic Review of Interventions (Version 6.1) [18], reported according to the Preferred Reporting Items for Systematic Reviews and Meta-analyses (PRISMA) 2013 statement [19]. The protocol has been registered with the international prospective register of systematic reviews, PROSPERO, and can be accessed with the registration code: CRD42020221657.

### 2.1. Data Sources and Search Strategies

Fourteen electronic databases (10 English: AMED, CINAHL, Cochrane Central, Embase, Emcare, MEDLINE, PubMed, ProQuest, Scopus, and Web of Science Core Collection and 4 Chinese: CNKI, CQVIP, Sinomed, and Wanfang Data) were comprehensively searched up to April 2021 with no language restrictions. Key search terms were synonyms of “overweight,” “obesity,” “Chinese herbal medicine,” and “phytotherapy.” A full electronic search strategy for the Ovid MEDLINE database is provided in Table S1. Results were filtered by the following criteria: RCTS, human, and adult. Hand search was performed by manually examining the bibliography of review articles that were retrieved.

### 2.2. Selection Criteria

RCTs were included if they (1) were conducted on adult participants with overweight or obesity; (2) administered CHM as an intervention (a major source of ingredients referenced in the Chinese pharmacopoeia [20]); (3) compared CHM treatment with placebo; (4) included one of total cholesterol, triglycerides, LDL cholesterol, HDL cholesterol as outcome measures; (5) applied parallel or crossover design (only first phase of crossover studies were considered if authors did not explicitly evaluate crossover effects); and (6) lasted ≥4 weeks. Cointervention was allowed if the same intervention regime was equally applied in both arms.

We excluded RCTs conducted on (1) children (aged ≤18), (2) a combination of healthy and overweight individuals, (3) interventions containing a single herbal ingredient only, (4) other forms of natural medicine (i.e., Ayurveda or homeopathy), or (5) outcome measures other than any of blood lipid profiles.

### 2.3. Data Extraction

Full-text RCTs were screened by two independent investigators (AW and ML). Findings extracted include study design: parallel or crossover, country of trial conduct, duration of study intervention, study sponsor, and population demographics: body weight, body mass index (BMI) and blood lipid profiles, age, gender, number of participants randomised, analysed, withdrawn, and reasons for withdrawal; intervention: treatment name, herbal ingredients, frequency, dosage, and cointerventions; the control: placebo name, ingredients, frequency, dosage, and cointerventions; and the included outcomes: total cholesterol, triglycerides, LDL cholesterol, and HDL cholesterol, with their respective summary statistics. Trial authors were contacted for missing information and confirmation of data. One reviewer (AW) entered the data of variables into the source database, while another reviewer (ML) validated the entry, and any discrepancies were resolved by consensus.

### 2.4. Quality Assessment

Cochrane risk-of-bias tool (RoB 2) was used to assess the quality of the included RCTs [18]. Rob 2 consists of five domains: the randomisation process, deviations from intended interventions, missing outcome data, measurement of the outcome, and selection of reported results. Each domain was assessed at the study level with outcomes of “low,” “high,” and “some concerns” based on various predefined signalling questions in RoB 2, where an algorithm was used to determine the final assessment for that domain. “High” risk of bias suggests that the methodology is likely to significantly affect the outcomes. “Some concern” either indicates insufficient reporting and lack of information and may affect the intervention to some extent. “Low” risk of bias implies that the method met the requirements of trial procedures and is not expected to significantly affect the outcome. We considered a trial implementing intention-to-treat (ITT) analysis if (1) it included all or most participants who were randomised or (2) it was stated in the methods where participants with at least one outcome measurement were included for analysis. Studies that excluded noncompliance were interpreted as per protocol analysis, even if it was not explicitly stated. Two reviewers (AW and ML) independently assessed the studies, and discrepancies were resolved by discussion and consensus.

### 2.5. Data Synthesis and Analysis

The primary analysis assessed the change in concentrations of total cholesterol, triglycerides, LDL cholesterol, and HDL cholesterol from baseline, obtained by subtracting the baseline from end of treatment values. Changes in outcomes were pooled using the generic inverse variance method fitted with a random-effects model, as this model yields a more conservative estimate compared to the fixed-effects model by accounting for within- and between-study heterogeneity [21, 22]. Continuous outcomes were expressed as mean difference (MD) with 95% confidence intervals (CIs), and categorical outcomes as risk ratio (RR) [18]. All analyses were two-tailed with a statistical significance of *p* < 0.05.

Heterogeneity between studies was assessed by the *I*^2^-statistic with statistical significance determined at *p* < 0.10 due to the presence of studies with no statistical significance [18]. Strategies employed to explore the sources of heterogeneity were sensitivity analyses, meta-regression, and subgroup analyses. Leave-one-out analysis and varying correlation coefficients (0.25 and 0.75) were performed to detect changes in the direction and magnitude of the effect estimate. Baseline measurements of total cholesterol, triglycerides, LDL cholesterol, HDL cholesterol, mean BMI, duration of the trial, the absence or presence of comorbidities, and the type of placebo intervention were analysed for potential modification of intervention effects. Publication bias was examined by funnel plots and quantified with Egger's regression and Begg's correlation tests. The “trim-and-fill” analysis was also performed to observe the impact of imputed studies on the overall effect estimates. A *p* value < 0.05 was considered significant for small-study effects. All statistical analyses and plots were conducted and generated in the R statistical environment using the “meta,” “metafor,” and “robvis” packages [23–25].

## 3. Results

### 3.1. Trial Characteristics

**Figure 1:**
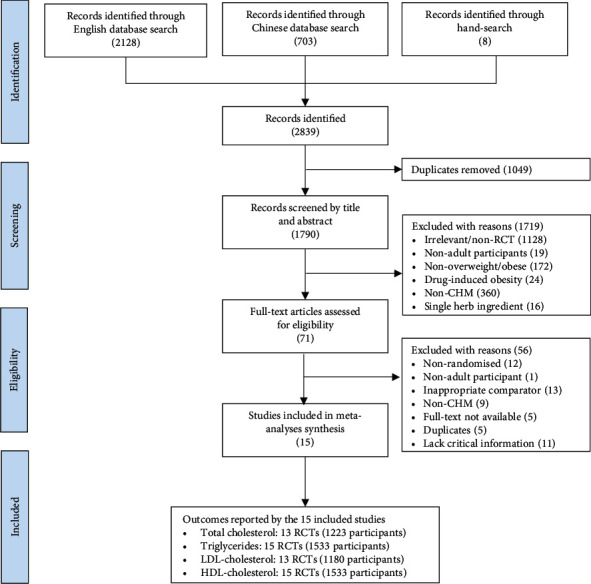
PRISMA flow diagram illustrating the procedures of literature search and screening of citations. CHM, Chinese herbal medicine, and RCT, randomised controlled trial.

A total of 2,839 citations were retrieved from the English database (2,128), Chinese database (703), and hand search (8). After removing duplicates and performing initial screening, 71 full texts were retrieved for further screening. Finally, 15 RCTs met the eligibility criteria and were included in the meta-analyses. The results of the literature search and screening process are illustrated in Figure 1. The characteristics of the included studies are summarised in Table 1.

The included RCTs, consisting of 1,533 participants, were conducted in China [34–40], Korea [26–28, 30, 33], Australia [32], Germany [29], and Japan [31]. Twelve studies recruited volunteers from an outpatient setting, two recruited from both out- and inpatients [37, 39], and one did not disclose such information [34]. The RCTs adopted a two-arm parallel design in a single-centre setting [27, 28, 30–32, 34–37, 39] or a multi-centre setting [26, 29, 33, 37, 40]. Body weight (>120%), BMI (>25 kg/m^2^ or >23 kg/m^2^ according to differing population cut-offs), and waist circumference (male >85 cm and female >80 cm) were indicative of diagnostic criteria for overweight or obesity in the included studies. The mean age of the participants was 45.17 years old, and 62.8% were female.

All included studies administered CHM as their primary treatment and placebo as their control (with or without lifestyle cointervention). The CHM formulae consist of herbs with the majority identified in the Chinese pharmacopoeia [20]. The placebo contains no active herbal ingredient (starch), 5% herbal decoction [35, 37], or 10% herbal decoction [40] used as a masking technique, not intended to induce detectable therapeutic benefits. Bioactive herbal compounds were validated in eight studies [26–32, 40], including three [27, 30, 40] that reported high-performance liquid chromatography (HPLC) techniques. Granule form [26, 31, 33, 34] was the most common, followed by decoction [27, 35, 40], capsule [30, 32, 39], and tablet [28, 29]. Among the 15 unique CHM formulae consisting of 61 herbal ingredients, the most frequently appearing herbs were *Rhei Rhizoma* (Da huang) and *Coptidis Rhizoma* (Huang lian); both administered in four different studies. Details of CHM formulae and corresponding placebos, including the ingredients and intervention regime, are documented in Table S2.

Calorie restriction or increased physical activity was implemented in 12 studies. Participants in these studies were (1) instructed to restrict their daily calorie intake [26, 28, 29, 33], (2) advised to have calorie reduction/consume a healthy diet [27, 34–37, 39], (3) asked to maintain their existing dietary habits [32, 40], or (4) had daily their energy intakes monitored [30, 31]. Regarding the exercise regime, participants were (1) instructed [39] or (2) advised [27, 34–37] to increase physical activity, (3) asked to maintain routine exercises [28, 29, 32, 33, 40], or (4) asked to have their activity status monitored [30, 31]. Various studies did not provide information on energy intake [38] and energy expenditure [26, 38].

All studies measured at least one of the blood lipid markers, including total cholesterol [26–35, 37, 38, 40], triglycerides [26–40], LDL cholesterol [26–32, 34, 35, 37–40], and HDL cholesterol [26–40], where 60% of studies reported in mmol/L. The units of analysis (mg/dL) were converted to mmol/L before meta-analysis was performed based on the standard formula (mean and direct subtraction; SD, based on Cochrane's Handbook using a correlation coefficient of 0.25, 0.5, and 0.75) [18]. Lipid markers were quantified using lipoprotein electrophoresis enzymes (total cholesterol, triglycerides, and LDL cholesterol) and ultracentrifugation receptor (HDL cholesterol) techniques.

The duration of the trials was between 4 weeks and 6 months, with a median of 12 weeks. Funding sources varied among the studies, ranging from not-for-profit agencies [27, 31–35, 37], industries [28, 29], and combined agency and industry [26, 40]; four RCTs did not disclose financial support [30, 36, 38, 39].

### 3.2. Risk of Bias Assessment

**Figure 2:**
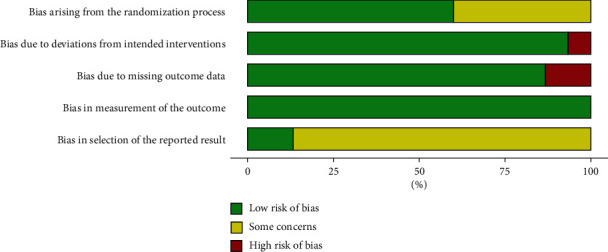
Risk-of-bias assessment summary of 15 included studies illustrating the distribution of “low” risk of bias, “some concerns,” and “high risk of bias” judgement across each domain. This boxplot was generated based on version 2 of the Cochrane risk-of-bias tool for randomised trials (RoB 2) using the “robvis” package in the R statistical environment.

After assessing clinical trial reports and protocols using Cochrane's RoB 2, only two [26, 33] of 15 studies were judged as “low” risk of bias throughout all 5 domains. The rest performed relatively well with “some concerns” in 2 major domains [27–32, 34–38], particularly in domain 1 “randomisation process” and domain 5 “selection of reported results.” It is worth noting that blood lipid profiles were generally the secondary outcomes of the included studies (80%) and were often neglected for full reporting in publications. The risk of bias summary is illustrated in Figure 2, and more details are provided in Figure S1.

#### 3.2.1. Randomisation Process

All studies stated that they were “randomised,” and 80% [26–34, 37, 39, 40] reported randomisation techniques (e.g., computer-generated codes and random number table). However, 53.3% [27, 28, 30–34, 37] lacked specification on how the allocation was concealed, thereby introducing “some concerns” for the influence of the investigator's knowledge in intervention assignment.

#### 3.2.2. Deviations from Intended Interventions

Of the 15 included studies, 4 were assessed as ITT [26, 29, 32, 33], while 11 were assessed as per protocol analysis. The overall risk of bias was assessed as “low” for 93.3% of studies, except for 1 assessed as “high” [40]. This study lacked information on participant flow and methods of analysis, and thus, “no information” on relevant signalling questions has led to a “high” risk of bias in this domain. Despite most studies blinding the participants relatively well by administering placebo with a similar appearance as treatment, a proportion (33.3%) [34, 36–38] of studies lacked sufficient reporting on the blinding of personnel delivering the assigned intervention.

#### 3.2.3. Missing Outcome Data

Overall, the included studies were assessed as “low” risk of bias (86.7%) for missing outcome data; however, two studies were assessed as “high” [39, 40]. The studies rated as “high” for missing outcomes have demonstrated drop-outs due to adverse events in the treatment group only, indicating the missing outcome may have depended on its true value. For studies that were unable to obtain data from at least 95% of randomised participants, either statistical analyses that corrected for bias were conducted or reasons for attrition were not influenced by its true value. Hence, most studies were rated as “low” for this domain.

#### 3.2.4. Measurement of the Outcome

All blood lipid profile outcomes were prespecified either in the registered protocols or in the methods section of published reports. As these outcomes were measured in an objective, nonjudgmental manner using comparable laboratory techniques at prespecified time points, it was therefore unlikely that bias was introduced in the outcome assessment process, compared to subjectively assessed outcomes. In this domain, all studies were rated as “low” risk of bias.

#### 3.2.5. Selection of the Reported Results

The majority of studies in this domain were assessed with “some concerns” (86.7%), with only two studies assessed as “low” risk of bias [26, 33]. The insufficient information regarding finalisation of the analysis before unblinding of outcome data and the lack of prospective specification for multiple analyses (baseline and end of treatment change) had contributed to “some concerns” on the selection of reported results in published articles.

### 3.3. Blood Lipid Outcomes

#### 3.3.1. Summary of Effects

The changes in blood lipid outcomes between and within intervention groups are listed in Table S3. All within-group changes of blood lipids from baseline were significant after CHM intervention (*p* < 0.05), while those of placebo were not significant except for the triglyceride outcome (*p* < 0.05). However, the pooled analysis revealed significant differences between the CHM and placebo groups for changes in triglycerides and HDL cholesterol outcomes at the end of treatment (*p* < 0.05).

#### 3.3.2. Effects of Total Cholesterol

**Figure 3:**
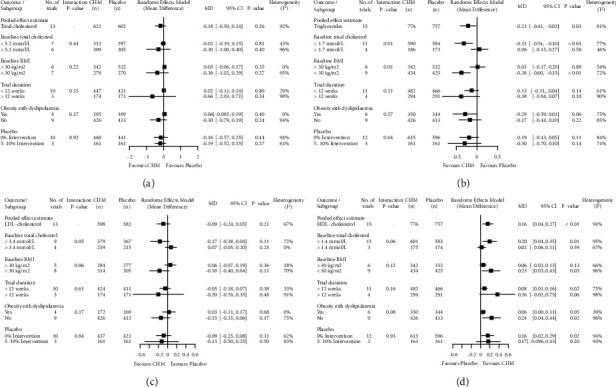
(a) Total cholesterol, (b) triglycerides, (c) LDL cholesterol, and (d) HDL cholesterol. Subgroup analysis of included trials investigating the effect of Chinese herbal medicine (CHM) and placebo on changes in HDL cholesterol. Data were pooled using the inverse variance method fitted with a random-effects model expressed as mean difference (MD) and 95% confidence intervals (CIs), and between-study variance was estimated with DerSimonian and Laird; two-tailed significance was set at a value of *p* < 0.05; *n*, number of participants.

The pooled result of 13 out of 15 studies (1,223 participants) comparing CHM with placebo is illustrated in Figure 3(a). Two studies lacked end of treatment or change values [36, 39]; therefore, they were not included in the meta-analysis. Although no significant differences in changes in total cholesterol were detected between the two groups, the effects favoured CHM treatment (MD −0.18 mmol/L, 95% CI −0.50 to 0.14, *I*2 = 92%). The leave-one-out analysis identified 1 outlier (Figure S2A) [38]; upon exclusion of the outlier, a substantial decline in heterogeneity was achieved, while the significance of intervention effects was not altered (MD −0.02 mmol/L, 95% CI −0.08 to 0.13, *I*^2^ = 18%; Figure S3A). Meta-regression analyses did not identify significant modifiers. Subgroup analyses for categorical covariates including total cholesterol at baseline, BMI at baseline, duration of the trial, obesity with dyslipidaemia, or type of placebo administered did not reveal any significant between-group differences (*p* > 0.05). However, there is a trend for increased effect sizes favouring the CHM group with baseline total cholesterol above 5.2 mmol/L, overweight participants with BMI <30 kg/m^2^, trial duration of more than 12 weeks, and participants with obesity without dyslipidaemia. Sensitivity analysis was performed using correlation coefficients of 0.25 and 0.75, and recalculation of effect estimates did not significantly alter the direction or magnitude of the pooled estimates (Table S4).

#### 3.3.3. Effects of Triglycerides

A total of 15 studies reported outcome values for triglycerides (1,533 participants), as seen in Figure 3(b). The pooled estimates indicated a significant reduction of triglyceride levels in the CHM group compared to placebo (MD −0.21 mmol/L, 95% CI −0.41 to −0.02, *I*^2^ = 81%). No significant outlier was detected in the leave-one-out analysis (Figure S2B). Continuous meta-regression identified baseline triglycerides and BMI as near-significant (*p* = 0.05) and significant modifiers (*p* = 0.01), accounting for 33.1% and 51.3% of residual heterogeneity, respectively. Categorical subgroup analyses revealed considerable between-study differences for baseline triglycerides (*p* = 0.01) and BMI (*p* = 0.01), where participants who were overweight and had higher baseline triglycerides contributed to greater effects of triglyceride reduction (BMI <30 kg/m^2^: MD −0.38 mmol/L, 95% CI −0.60 to −0.15, *I*^2^ = 72%; baseline triglycerides >1.7 mmol/L: MD −0.32 mmol/L, 95% CI −0.54 to −0.10, *I*^2^ = 77%). Furthermore, sensitivity analyses using various correlation coefficients (0.25 and 0.75) did not result in a change in significance or direction of the effect estimate (Table S4).

#### 3.3.4. Effects of LDL Cholesterol

The forest plot in Figure 3(c) shows 13 of the 15 studies (1,180 participants) included in the pooled analysis for the LDL cholesterol outcome, where two lacked the end of treatment or change values [33, 36]. Despite the effect estimate being in favour of the CHM group for lowering LDL levels compared to placebo, a statistically significant difference was not achieved (MD −0.09, 95% CI −0.24 to 0.05, *I*^2^ = 67%). While the exclusion of an outlying study [38] identified from leave-one-out analyses revealed similar magnitude and direction of intervention effects (MD −0.02, 95% CI −0.13 to 0.09, *I*^2^ = 34%), it resulted in a substantial drop of heterogeneity from 67% to 34% (Figures S3B and S2C). Both meta-regression and subgroup analyses of covariates did not identify significant differences. However, a lower LDL cholesterol (<3.4 mmol/L) and BMI (<30 kg/m^2^), a trial duration of more than 12 weeks, and participants with obesity without dyslipidaemia had contributed to a trend indicating a stronger effect size in favour of the CHM group. Moreover, the intervention effects of CHM and placebo on the change in LDL levels were consistently observed across the use of 0.25 or 0.75 correlation coefficients (Table S4).

#### 3.3.5. Effects of HDL Cholesterol

The forest plot in Figure 3(d) demonstrates the 15 included studies that reported outcomes for HDL cholesterol (1,533 participants). A substantial increase from baseline in HDL cholesterol was detected favouring the CHM group compared to the placebo (MD 0.16 mmol/L, 95% CI 0.04 to 0.27, *I*^2^ = 94%). Furthermore, an outlying study [38] was identified based on leave-one-out analysis (Figure S2D). After excluding the outliers, a slight decline of heterogeneity and no considerable change in the direction of intervention effects were observed (MD 0.08 mmol/L, 95% CI 0.03 to 0.14, *I*^2^ = 75%; Figure S3C). For subgroup analysis, between-study heterogeneity was not substantial, although baseline HDL cholesterol as a covariate approached significance (*p* = 0.06). Besides, there was a trend for increased effect sizes favouring CHM intervention for participants who had a lower baseline HDL cholesterol and baseline BMI, were overweight without dyslipidaemia, participated in a trial lasting more than 12 weeks, and had 5–10% of placebo intervention. However, these subgroup differences were not statistically significant. Furthermore, the recalculation of effect estimates using correlation coefficients of 0.25 or 0.75 did not affect the direction and significance of the pooled HDL cholesterol outcomes (Table S4).

#### 3.3.6. Safety Outcomes

*(1) Attrition Rates*. All 15 studies provided information on drop-outs, including 5 reporting that all participants completed the study [35–39]. For the remaining 10 studies, a lower attrition rate favoured participants in the CHM group; however, a significant reduction in risk ratio was not achieved (RR 0.83, 95% CI 0.69 to 1.01, *I*^2^ = 0%).

*(2) Safety Parameters*. Twelve (80%) studies systematically assessed and monitored safety parameters such as vital signs or biochemical profiles [26–29, 32–35, 37–40]. Of these, all except for one [33] declared no clinically significant deviation of safety outcomes at the end of the study (*p* > 0.05). A significant change in aspartate transaminase (AST) and alanine transaminase (ALT) in the treatment group was detected in one study [33]; however, authors declared that the changes were within clinically normal ranges.

*(3) Adverse Events*. All studies (93.3%) except for one [38] reported information on the frequency of adverse events. Three studies reported no adverse events in the treatment group [34, 35, 39], and two lacked appropriate information for synthesis [27, 36]. Based on good coding practices [18], two studies [26, 27] further classified adverse events according to MedDRA and CTCAE terminology, respectively. The risk ratio for the frequency of adverse events was not significantly higher in the CHM group than the placebo group based on the pooled estimate of the remaining nine studies (RR 2.44, 95% 0.94 to 6.36, *I*^2^ = 80%). Furthermore, it is encouraged that trial authors provide insights on whether or not they believed the adverse events were associated with the administration of treatment intervention [18]. Authors in two studies (12.5%) [31, 33] contributed to this information, where one indicated a direct association of the adverse event with a specific herbal ingredient and another had ruled out treatment-related adverse events.

**Figure 4:**
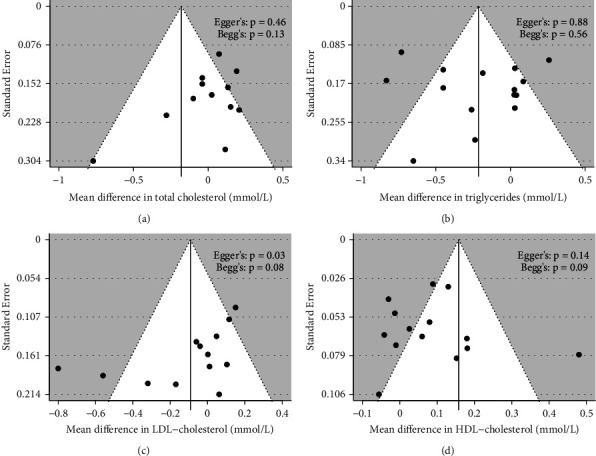
Funnel plots of change from baseline total cholesterol (a), triglycerides (b), LDL cholesterol (c), and HDL cholesterol (d) outcomes. The pooled effect estimate (mean difference) is represented by a solid line, framed by the dotted pseudo 95% confidence limits. Egger's regression test was performed using standard error prediction using the inverse variance method fitted to a random-effects model, while between-study variance was obtained with DerSimonian and Laird estimators. Significance was set at *p* < 0.05.

*(4) Publication Bias*. Visual inspection of the funnel plots revealed potential asymmetry for most outcomes except for triglycerides (Figure 4). However, while this observation was further quantified in Egger and Begg's tests, a significant influence from small studies only occurred in LDL cholesterol outcome reflected in Egger's regression test (Egger's: *p* = 0.03, Begg's: *p* = 0.08). Trim-and-fill analyses for total cholesterol, triglycerides, LDL cholesterol, and HDL cholesterol outcomes identified 4, 1, 2, and 5 missing studies, respectively, which may mitigate publication bias (Figure S4). With the inclusion of 4 imputed studies for total cholesterol outcome and 2 for LDL cholesterol outcome, the intervention effects were significantly altered (total cholesterol: MD −0.34 mmol/L, 95% CI −0.63 to −0.05, *I*^2^ = 92%; LDL cholesterol: MD −0.15, 95% CI −0.30 to −0.001, *I*^2^ = 76%). In contrast, the significance favouring CHM treatment for triglycerides and HDL cholesterol remained consistent with the inclusion of 1 and 5 imputed studies, respectively (Figure S4).

## 4. Discussion

This systematic review and meta-analysis comprehensively searched published RCTs on the clinical effects of CHM versus placebo on lipid metabolism biomarkers among adults with overweight or obesity. Findings of 15 included studies (1,533 participants) revealed that CHM may provide significant lowering of triglycerides and HDL cholesterol concentrations at the end of treatment compared to placebo. Our analyses also showed that reductions in total cholesterol and LDL cholesterol levels were not significant but favoured CHM interventions among 13 of the 15 included studies that reported these outcomes. Furthermore, meta-regression and subgroup analyses identified substantial between-group differences for baseline triglycerides and BMI variables, suggesting that the lipid-lowering effect is more pronounced after CHM treatment among overweight participants with hypertriglyceridemia at baseline.

Furthermore, our findings were consistent with the existing herbal medicine reviews. A published meta-analysis of 1,112 dyslipidaemia and diabetic patients reported significantly lowered total cholesterol, triglycerides, and LDL cholesterol by 0.52, 0.21, and 0.21 mmol/L, respectively, in the CHM treatment group compared to placebo or Western medication at the end of treatment [9]. Greater reductions in these parameters by 0.88, 0.71, and 0.74 mmol/L post-CHM interventions were demonstrated in another meta-analysis conducted by Qiao et al. [10] on 1,357 dyslipidaemia participants. The stronger effect estimates in Qiao et al. may be attributed to (1) the narrower inclusion criteria targeting dyslipidaemia participants, (2) the evaluation of CHM interventions on one specific herbal ingredient (i.e., *Nelumbinis Folium*, He Ye), and (3) the use of change-from-baseline values to calculate effect estimates. Although improvements in HDL cholesterol favouring CHM were observed in both reviews, they were not statistically significant. Our study, on the other hand, demonstrated a modest but significant increase of 0.16 mmol/L (13%) following CHM treatment. This could be due to the relatively small confidence interval that could allow for more precise estimates of HDL cholesterol among the population in our included trials. Despite the differences in population demographics, intervention regimes, and level of comparators, the magnitude and direction of CHM intervention effects on triglycerides echoed the findings in our review.

Based on the joint European Society of Cardiology (ESC)/European Atherosclerosis Society (EAS) guidelines [5], the primary effects of statins significantly lower LDL cholesterol, and add-on effects typically reduce triglycerides by 10–20% and elevate HDL cholesterol by 1–10%. The present study suggests that CHM may decrease triglycerides by 0.21 mmol/L (11%) and increase HDL cholesterol by 0.16 mmol/L (13%) compared to placebo over a median of 12 weeks, which is within the clinical guidelines of ESC/EAS for typical statin therapy. As new treatments for dyslipidaemia such as cholesterol absorption inhibitors, bile acid sequestrants, proprotein convertase subtilisin/kexin type 9 (PCSK9) inhibitors, and fibrates are emerging [5, 41], future meta-analyses on CHM compared with various classes of lipid-lowering medications are worthy to establish an understanding of their roles in lipid metabolism.

Most interventions were well tolerated, and no serious adverse events nor clinically significant abnormalities in kidney and liver function were reported in the CHM treatment group based on available data. Reported symptoms were mild to moderate gastrointestinal events such as loose bowels, increased flatulence, gastric pain, nausea, diarrhoea, and constipation. Some of these events have been attributed to the actions of herbal medicines on the gut microbiota for ameliorating metabolic conditions [42]. In addition, mild to moderate neurological symptoms such as headache and dizziness were identified [26–28, 32]. These haemodynamic events may have been due to differing tolerability thresholds for certain compounds derived from *Camellia sinensis* (*Cha Ye*) and *Ephedra sinica* (*Ma Huang*), where symptoms have been linked to higher dosing concentrations [43]. It is therefore crucial to quantify the active ingredients contained in CHM formulations based on the pharmaceutical index to ensure the safe administration of herbal medicines [44]. As participants with obesity may be at high risk of developing comorbidities, indicators such as vital signs, glucose metabolism, and kidney/liver function should also be closely monitored throughout the intervention and follow-up periods. Failure to report sufficient details on these indicators has limited our ability to synthesise data. Future trials may consider the use of internationally recognised codes such as the Medical Dictionary for Regulatory Activities (MedDRA) and the Common Terminology Criteria for Adverse Events (CTCAE) for more precise reporting of adverse events [45, 46].

Among the 15 unique formulae included in this study, *Coptidis Rhizome* and *Rhei Rhizoma* appeared most frequently used in four different preparations. A remarkably improved plasma concentration of blood lipid outcomes compared to placebo or no treatment was revealed in a meta-analysis of 497 participants who were administered berberine, a bioactive compound isolated from *Coptidis Rhizome* [11]. Furthermore, a combination of berberine with other alkaloids of *Coptidis Rhizome*, including coptisine, palmatine, epiberberine, and jatrorrhizine, may produce synergistic effects to modulate lipid absorption, synthesis, and metabolism [47, 48]. When administered concurrently, these compounds provided more potent cholesterol elimination effects by upregulating LDL receptor and 7-alpha-hydroxylase (CYP7A1) expression in diet-induced hyperlipidaemic animal models [49]. Restricting hepatic 3-hydroxy-3-methylglutaryl-CoA reductase (HMGCR) expression by berberine and palmatine has also been shown to impede cholesterol esterification and synthesis [49, 50]. In addition, key anti-lipidaemic anthraquinones derived from *Rhei Rhizoma* such as chrysophanol provided significant fatty acid oxidation, lipolysis, and upregulation of thermogenesis gene expression in 3T3-adipocytes [15]. These improvements were also observed in obese mice. Chrysophanol may alleviate obesity and attenuate triglyceride accumulation by promoting lipolysis through mediating adenosine 5-monophosphate activated protein kinase (AMPK) and hormone-sensitive lipase (HSL) pathways [15, 51]. Taken together, these active ingredients could be major contributors to the lipid modulating effects in formulations included in our review, and their cellular mechanisms could be further elucidated in computational studies.

Methodological quality plays a crucial role in the strength of evidence and adhering to rigorous protocols with sufficient reporting may provide greater credibility of findings. Information on allocation concealment and blinding of assessors were lacking in 50% and 80% of the included studies, respectively. However, the objective nature of blood lipid outcome is not expected to cause any deviation from intended interventions nor to provide overestimated effects compared to subjectively assessed outcomes [52]. Additionally, the inconsistent reporting of missing data is common a concern evident in interventional trials [53]. Based on the RoB 2 signalling question 3.1, many high-quality clinical trials were unable to achieve desirable thresholds of analysing data from 95% of randomised participants [26, 32]. As the ITT analysis adopts various imputation methodologies, it is essential for trial authors to clearly define the corresponding statistical procedures, where applicable, to aid the large-scale synthesis of data. Furthermore, a lower risk of selective reporting bias may be achieved by prospective registration of trial protocols on publicly accessible databases [54]. Registration was apparent in 46% of the included studies, although the majority lacked methods of statistical analysis and time point of measurement. Thus, we were unable to rule out potential biases in selective analysis and reporting of data in included trials. Pre-registrations with sufficient details are encouraged in future studies.

Methodological variations in participants' health condition at baseline, herbal preparations, and trial duration were among the major contributors of heterogeneity in this study. Although our study attempted to limit control interventions to placebo as current research found no evidence of clinically significant effects on placebo interventions [55], heterogeneity remained high (*I*^2^ >50%). Therefore, subgroup analyses and meta-regression were performed to explore the sources of heterogeneity. Based on our predefined subgroups, between-group effects were greater for baseline triglycerides beyond normal levels (>1.7 mmol/L) and among overweight participants (BMI <30 kg/m^2^), although their respective distributions were nonlinear. No statistically significant differences in blood lipid outcomes among subjects with obesity may indicate that conventional therapy is necessary for the effective management of high-risk participants. Although four studies administered 5–10% of decoction in the placebo to aid masking, there were no differences between these studies and pharmacologically inert applications. This suggests that extremely low doses of active ingredients below therapeutic thresholds may not exert significant clinical effects. However, the heterogeneity among studies with inert or diluted decoction was high, suggesting the need for further research in this area [56]. Furthermore, the improvements in triglycerides and HDL cholesterol post-CHM administration were not significantly altered following sensitivity analyses, implying the presence of considerable robustness and reliability of findings. Additionally, fitting all analyses in a random-effects model accounts for between-study heterogeneity and is thought to produce more conservative estimates when small study effects were nonsubstantial, as is observed in our meta-analysis [18]. Lastly from the subgroup analyses, an interesting observation on clinical effects based on trial duration emerged. There was a wide confidence interval and a high *I*^2^ among long-term trials (>12 weeks), consistently observed in all four outcome measures, in contrast to short-term trials (<12 weeks). These did not allow for conclusive findings, and thus, future reviews could focus on trials with a duration of >12 weeks to examine the long-term effects of CHM interventions on blood lipid profiles.

Nevertheless, this review had several strengths. This study is one of the first systematic reviews to examine the role of herbal formulations against placebo in modulating blood lipid profiles. Second, it overcomes the generalisability of results from existing reviews conducted on populations with differing body compositions and provides insights specifically for overweight and obese participants. As overweight and obese participants were reported to have altered metabolic pathways particularly in lipid and glucose metabolism, a more consistent clinical effect of herbal medicines on total cholesterol, triglycerides, LDL cholesterol, and HDL cholesterol could be observed. In addition, there are significant limitations worth noting. Firstly, covariates including macronutrient intake, physical activity, alcohol consumption, and smoking status could be investigated as these covariates have been reported to significantly affect lipid metabolism [57, 58]. Secondly, incomplete reporting of evidence may have contributed to underpowered results, particularly for total and LDL cholesterol outcomes, as “trim-and-fill” analysis incorporating more studies exhibited significant differences favouring CHM treatment. Third, this review is limited to the four main lipid outcomes. Other clinically relevant lipoprotein biomarkers (apolipoprotein A and B), and proinflammatory adipokines [5] may be examined for a more comprehensive understanding of CHM effects on lipid metabolism and cardiovascular risk among participants with obesity.

As one of the aims of this systematic review is to provide an understanding of the roles of herbal formulations on blood lipid profiles among weight management trials, a meta-analysis was conducted for this purpose. Studies with different herbal formulations were synthesised to provide insights regarding their relative strengths on blood lipid profiles, consistent with approaches used in recent Cochrane reviews on herbal medicines [59]. Although there was heterogeneity in trial methodologies, particularly the herbal composition, dosages, and treatment duration, this study fulfilled its aim as a reference guide with a summary of current Chinese herbal formulations on total cholesterol, triglycerides, LDL cholesterol, and HDL cholesterol compared to placebo. This study also highlighted specific formulations with possible efficacy and encouraged further investigation in long-term, large-scale, and rigorously conducted randomised controlled trials.

In summary, this meta-analysis demonstrated that CHM formulations administered in weight-loss studies could improve triglycerides and HDL cholesterol levels over a median of 12 weeks. Interventional effects were more pronounced in overweight subjects with higher triglyceride concentrations at baseline; however, the distribution was nonlinear. No significant adverse events or attrition rates were observed between CHM and placebo groups within the treatment period, suggesting that the interventions were well tolerated. Although findings tend to favour CHM, careful interpretation is required as trials were conducted on relatively small populations with short durations. The incomplete reporting of critical information also limited the synthesis of trial data. These issues could be considered in the future design of CHM trials.

## Figures and Tables

**Table 1:** Characteristics of 15 included studies.

Author		*n*	Gender	BMI (kg/m^2^)	Age (years)	Setting	Site, §	Wks	Characteristics	Diagnosis	Interventions	Drop-out	RoB^¶^	Source
Cheon et al. [26]	I	76	0 M; 76 F	32.4 (31.6, 33.3) †	43.7 (41.6, 45.8) †	OP	MC, KOR	12	Hyperlipidaemia, hypertension, obese, overweight, T2DM	≥27 kg/m^2^	Euiiyin-tang	14	L	A-D
	C	73	0 M; 73 F	33.3 (32.3, 34.3) †	42.5 (40.0, 44.9) †						Placebo	13		

Cho et al. [27]	I	39	3 M; 27 F	28.35 (3.95)	42.9 (12.67)	OP	SC, KOR	∼8	Obese, overweight	≥23 kg/m^2^	THI	9	S	A
	C	30	5 M; 18 F	26.51 (2.21)	41.83 (14.82)						Placebo	7		

Cho et al. [28]	I	30	10 M; 20 F	27.1 (1.5)	39.5 (11.2)	OP	SC, KOR	12	Overweight	25.0–29.9 kg/m^2^	YY-312	10	S	D
	C	30	8 M; 22 F	27.2 (1.2)	41.7 (11.1)						Placebo	11		

Chong et al. [29]	I	46	12 M; ∼34 F	28.5 (2.1)	43.1 (10.8)	OP	MC, DEU	12	Obese, overweight	25–32 kg/m^2^	IQP-GC-101	4	S	D
	C	46	17 M; ∼29 F	28.6 (1.8)	42.5 (11.6)						Placebo	4		

Chung et al. [30]	I	13	6 M; 4 F	29.5 (3.63)	50 (5.85)	OP	SC, KOR	8	Hyperlipidaemia, hypertension, obese, overweight, prediabetic	≥25 kg/m^2^	QXD	3	S	NR
	C	13	6 M; 4 F	28.89 (2.96)	45.2 (9.52)						Placebo	3		

Hioki et al. [31]	I	44	0 M; 41 F	36.7 (6.8)	52.6 (14)	OP	SC, JPN	24	Obese, prediabetic	NR	Bofu-tsusho-san	3	S	A
	C	41	0 M; 40 F	36.1 (3.3)	54.8 (12.5)						Placebo	1		

Lenon et al. [32]	I	59	10 M; 49 F	35.3 (4.8)	39.3 (13.2)	OP	SC, AUS	12	Obese, T2DM, controlled hypertension	≥30 kg/m^2^	RCM-104	13	S	A
	C	58	10 M; 48 F	36 (5.5)	40.4 (10.2)						Placebo	17		

Park et al. [33]	I	58	7 M; 50 F	31.8 (2.6)	39.2 (9.5)	OP	MC, AUS	12	Hyperlipidaemia, hypertension, obese, overweight, T2DM	≥27 kg/m^2^	TJ001	10	L	A
	C	55	10 M; 45 F	31.9 (3.8)	38.8 (10.1)						Placebo	17		

Sheng et al. [34]	I	35	10 M; 24 F	31.68 (2.87)	37.74 (12.39)	OP	SC, CHN	∼4	Obese	≥28 kg/m^2^	Jianpi Shugan Jiangzhifang Fang	1	S	A
	C	35	13 M; 21 F	31.77 (4.07)	39.29 (10.11)						Placebo	1		

Sun et al. [35]	I	47	22 M; 25 F	26.54 (2.685)	55.21 (8.03)	OP	SC, CHN	12	Obese, T2DM, overweight, diabetic nephropathy, diabetic optic retinopathy	≥24 kg/m^2^	Yiqi huatan huoxue zhongyao Fufang		S	A
	C	51	23 M; 28 F	26.42 (2.64)	55.44 (7.96)						5% intervention			

Tang [36]	I	120	78 M; 42 F	25.91 (0.68)	53 (45, 70)	OP	SC, CHN	∼26	MetS (obese, overweight, T2DM, hypertension, dyslipidaemia)	≥25 kg/m^2^	Soufeng Shunqi Wan	0^*∗*^	S	NR
	C	120	75 M; 45 F	25.89 (0.75)	52 (44, 72)						Placebo	0^*∗*^		

Wang et al. [37]	I	60	NR	27.78 (2.41)	50.11 (9.96)	IP, OP	MC, CHN	12	MetS (T2DM, hyperlipidaemia, obese, hypertension)	*M* > 90 cm; *F* > 85 cm	Yiqi huaju Fang	0^*∗*^	S	A
	C	60	NR	27.74 (2.19)	51.97 (9.39)						5% intervention	0^*∗*^		

Wang et al. [38]	I	48	28 M; 20 F	∼24	49.69 (NR)	OP	SC, CHN	∼26	Obese, overweight	≥24 kg/m^2^; *M* ≥ 85 cm, *F* ≥ 80 cm	Jianfei heji	0^*∗*^	S	NR
	C	48	27 M; 21 F	∼24	52.23 (NR)						Placebo	0^*∗*^		

Xu [39]	I	∼35	17 M; 13 F	29.5 (3.1)	46 (12.2)	IP, OP	SC, CHN	12	Dyslipidaemia, hypertension, hyperuricaemia, obese, overweight, T2DM	BMI>26 kg/m^2^; BW >120% of standard weight; BF > 130%	Hefeiqi Jiaonang		H	NR
	C	∼35	16 M; 14 F	28.9 (3.8)	46 (9.3)						Placebo			

Zhou et al. [40]	I	70	31 M; 39 F	33.02 (3.47)	39.91 (11.5)	OP	MC, CHN	24	Obese, overweight	28–39.99 kg/m^2^; *M* ≥ 85 cm, *F* ≥ 80 cm	Xin Jiu Xiao Gaofang	11	H	A-D
	C	70	29 M; 35 F	33.42 (3.73)	40.02 (11.98)						10% intervention	15		

∼ indicates that results were estimated based on available evidence within the reports; A = agency including government, university, or not-for-profit agency; A-D = a combined source of agency and industry; BF, body fat (%); BMI, body mass index (kg/m^2^); BW, body weight (kg); C, control group; D = industry including pharmaceutical companies or business entities; F, female; I, intervention group; IP, in-patient; M, male; MC, multi-centre trial site; n, number of participants; OP, out-patient; RoB, risk of bias; SC, single-centre trial site; T2DM, type 2 diabetes mellitus. ^†^Cheon et al [26] presented results in mean and 95% confidence intervals. ^§^Country of trial conduct was represented by the International Standard for country codes (ISO 3166-3). ^¶^Assessed based on the version of the Cochrane's risk-of-bias tool, overall RoB score was presented: “L” represents low risk of bias; “S” represents some concerns; “H” represents high risk of bias.

## Data Availability

Data generated from this systematic review are included within the manuscript and the supplementary materials. Additional data are available upon request from the corresponding author.

## Supplementary Materials

Supplementary Materials Contents of the supplementary file include the following tables and figures. Table S1: search strategy example used to retrieve citations from OVID MEDLINE database. Table S2: details of CHM and placebo intervention regimes. Table S3: comparison of changes in blood lipid profiles within and between CHM and placebo groups. Table S4: sensitivity analysis of 15 included study showing recalculation of effect estimates with correlation coefficients of 0.25, 0.50 (main meta-analysis), and 0.75. Figure S1: risk of bias summary of individual studies included in the meta-analysis based on Cochrane's Risk of Bias 2 Tool. Figure S2: leave-one-out analysis to detect various outliers for total cholesterol, triglyceride, LDL cholesterol, and HDL cholesterol outcomes. Figure S3: forest plots after significant outlier removed for total cholesterol, LDL cholesterol, and HDL cholesterol outcomes. Figure S4: funnel plots with trim-and-fill analysis for change from baseline total cholesterol, triglyceride, LDL cholesterol, and HDL cholesterol outcomes.

## References

[1] Vekic J., Zeljkovic A., Stefanovic A., Jelic-Ivanovic Z., Spasojevic-Kalimanovska V. (2019). Obesity and dyslipidemia. *Metabolism*.

[2] Koene R. J., Prizment A. E., Blaes A., Konety S. H. (2016). Shared risk factors in cardiovascular disease and cancer. *Circulation*.

[3] Bays H. E., Toth P. P., Kris-Etherton P. M. (2013). Obesity, adiposity, and dyslipidemia: a consensus statement from the National Lipid Association. *Journal of Clinical Lipidology*.

[4] Cicero A. F. G., Landolfo M., Ventura F., Borghi C. (2019). Current pharmacotherapeutic options for primary dyslipidemia in adults. *Expert Opinion on Pharmacotherapy*.

[5] Mach F., Baigent C., Catapano A. L. (2019). ESC/EAS Guidelines for the management of dyslipidaemias: lipid modification to reduce cardiovascular risk. *European Heart Journal*.

[6] Newman C. B., Preiss D., Tobert J. A. (2019). Statin safety and associated adverse events a scientific statement from the American heart association. *Arteriosclerosis, Thrombosis, and Vascular Biology*.

[7] Luo S., Lenon G. B., Gill H. (2019). Do the natural chemical compounds interact with the same targets of current pharmacotherapy for weight management?-A review. *Current Drug Targets*.

[8] Ji X., Shi S., Liu B. (2019). Bioactive compounds from herbal medicines to manage dyslipidemia. *Biomedicine & Pharmacotherapy*.

[9] Wang H., Wang J., Hou Y. (2020). Clinical efficacy and safety of traditional Chinese medicine in treatment of dyslipidaemia: a meta-analysis [Chinese]. *Chinese Architecture Traditional Chinese Medicine*.

[10] Qiao Y., Zhang J., Ma L., Lei S. (2018). Efficacy of Folium Nelumbinis on dyslipidaemia: a meta-analysis. *Chinese Journal of Integrative Medicine on Cardio-/Cerebrovascular Disease*.

[11] Ju J., Li J., Lin Q., Xu H. (2018). Efficacy and safety of berberine for dyslipidaemias: a systematic review and meta-analysis of randomized clinical trials. *Phytomedicine*.

[12] Onakpoya I., Spencer E., Heneghan C., Thompson M. (2014). The effect of green tea on blood pressure and lipid profile: a systematic review and meta-analysis of randomized clinical trials. *Nutrition, Metabolism and Cardiovascular Diseases*.

[13] Hernández-García D., Granado-Serrano A. B., Martín-Gari M., Naudí A., Serrano J. C. (2019). Efficacy of Panax ginseng supplementation on blood lipid profile. A meta-analysis and systematic review of clinical randomized trials. *Journal of Ethnopharmacology*.

[14] Gadde K. M., Martin C. K., Berthoud H.-R., Heymsfield S. B. (2018). Obesity. *Journal of the American College of Cardiology*.

[15] Xie L., Tang H., Song J., Long J., Zhang L., Li X. (2019). Chrysophanol: a review of its pharmacology, toxicity and pharmacokinetics. *Journal of Pharmacy and Pharmacology*.

[16] Koppen L. M., Whitaker A., Rosene A., Beckett R. D. (2017). Efficacy of berberine alone and in combination for the treatment of hyperlipidemia: a systematic review. *Journal of Evidence-Based Complementary & Alternative Medicine*.

[17] Liao J., Tian J., Li T., Song W., Zhao W., Du J. (2014). Xuefuzhuyu decoction for hyperlipidemia: a systematic review and Meta-analysis of randomized clinical trails. *Journal of Traditional Chinese Medicine*.

[18] Higgins J. P. T., Thomas J., Chandler J. (2019). *Cochrane Handbook for Systematic Reviews of Interventions*.

[19] Moher D., Liberati A., Tetzlaff J., Altman D. G. (2009). Preferred reporting items for systematic reviews and meta-analyses: the PRISMA statement. *Journal of Clinical Epidemiology*.

[20] Chinese Pharmacopoeia Commission (2015). *Pharmacopoeia of the People’s Republic of China Part I*.

[21] Schmidt F. L., Oh I.-S., Hayes T. L. (2009). Fixed- versus random-effects models in meta-analysis: model properties and an empirical comparison of differences in results. *British Journal of Mathematical and Statistical Psychology*.

[22] Hunter J. E., Schmidt F. L. (2000). Fixed effects vs. Random effects meta‐analysis models: implications for cumulative research knowledge. *International Journal of Selection and Assessment*.

[23] McGuinness L. A., Higgins J. P. T. (2020). Risk‐of‐bias VISualization (robvis): an R package and Shiny web app for visualizing risk‐of‐bias assessments. *Research Synthesis Methods*.

[24] Balduzzi S., Rücker G., Schwarzer G. (2019). How to perform a meta-analysis with R: a practical tutorial. *Evidence-Based Mental Health*.

[25] Viechtbauer W. (2010). Conducting meta-analyses in R with the metafor package. *Journal of Statistical Software*.

[26] Cheon C., Song Y.-K., Ko S.-G. (2020). Efficacy and safety of Euiiyin-tang in Korean women with obesity: a randomized, double-blind, placebo-controlled, multicenter trial. *Complementary Therapies in Medicine*.

[27] Cho S. H., Yoon Y., Yang Y. (2013). The evaluation of the body weight lowering effects of herbal extract THI on exercising healthy overweight humans: a randomized double-blind, placebo-controlled trial. *Evidence-Based Complementary and Alternative Medicine*.

[28] Cho Y.-G., Jung J.-H., Kang J.-H., Kwon J. S., Yu S. P., Baik T. G. (2017). Effect of a herbal extract powder (YY-312) from Imperata cylindrica Beauvois, Citrus unshiu Markovich, and Evodia officinalis Dode on body fat mass in overweight adults: a 12-week, randomized, double-blind, placebo-controlled, parallel-group clinical trial. *BMC Complementary and Alternative Medicine*.

[29] Chong P. W., Beah Z. M., Grube B., Riede L. (2014). IQP‐GC‐101 reduces body weight and body fat mass: a randomized, double‐blind, placebo‐controlled study. *Phytotherapy Research*.

[30] Chung W., Ryu J., Chung S., Kim S. (2016). Effect of Qingxue Dan on obesity and metabolic biomarker: a double-blind randomized-controlled pilot study. *Journal of Traditional Chinese Medicine*.

[31] Hioki C., Yoshimoto K., Yoshida T. (2004). Efficacy of bofu-tsusho-san, an oriental herbal medicine, in obese Japanese women with impaired glucose tolerance. *Clinical and Experimental Pharmacology and Physiology*.

[32] Lenon G. B., Li K. X., Chang Y.-H. (2012). Efficacy and safety of a Chinese herbal medicine formula (RCM-104) in the management of simple obesity: a randomized, placebo-controlled clinical trial. *Evidence-Based Complementary and Alternative Medicine*.

[33] Park S., Nahmkoong W., Cheon C. (2013). Efficacy and safety of taeeumjowi-tang in obese Korean adults: a double-blind, randomized, and placebo-controlled pilot trial. *Evidence-Based Complementary and Alternative Medicine*.

[34] Sheng Z., Hu Y., Liu J., Ying R., Shen J. (2017). Effect of JianPi ShuGan JiangZhi formula on simple obesity and the expression of leptin and adiponectin [Chinese]. *World Chinese Medicine*.

[35] Sun L., Tang X., Zhang P. (2017). Clinical observation on method of supplementing Qi, resolving phlegm and activating blood circulation in improving disorder of glucose and lipid metabolism and obestatin in overweight/obese of type 2 diabetes mellitus patients [Chinese]. *Chinese Journal of Experimental Traditional Medical Formulae*.

[36] Tang D. (2007). Soufeng Shunqi pills for metabolic syndrome in 120 patients [Chinese]. *Shandong Journal of Traditional Chinese Medicine*.

[37] Wang T., Huo Q., Fu X., He Y., Wang W. (2016). Treating type 2 diabetes mellitus patients complicated with metabolic syndrome by benefiting Qi dissolving [Chinese]. *Chinese Journal of Integrated Traditional and Western Medicine*.

[38] Wang Q., Lin Z., Lu J. (2007). Clinical observation of self-made weight-loss formulation for simple obesity [Chinese]. *Clinical Journal of Traditional Chinese Medical*.

[39] Xu H. (2008). Clinical observation on method of regulating triple burner for simple obesity [Chinese].

[40] Zhou Q., Chang B., Chen X.-Y. (2014). Chinese herbal medicine for obesity: a randomized, double-blinded, multicenter, prospective trial. *The American Journal of Chinese Medicine*.

[41] Banach M., Jankowski P., Jóźwiak J. (2017). PoLA/CFPiP/PCS guidelines for the management of dyslipidaemias for family physicians 2016. *Archives of Medical Science*.

[42] Tong X., Xu J., Lian F. (2018). Structural alteration of gut microbiota during the amelioration of human type 2 diabetes with hyperlipidemia by metformin and a traditional Chinese herbal formula: a multicenter, randomized, open label clinical trial. *MBio*.

[43] Temple J. L., Bernard C., Lipshultz S. E., Czachor J. D., Westphal J. A., Mestre M. A. (2017). The safety of ingested caffeine: a comprehensive review. *Frontiers in Psychiatry*.

[44] Cheng C.-W., Wu T.-X., Shang H.-C. (2017). CONSORT extension for Chinese herbal medicine formulas 2017: recommendations, explanation, and elaboration. *Annals of Internal Medicine*.

[45] Trotti A., Colevas A. D., Setser A., Basch E. (2007). Patient-reported outcomes and the evolution of adverse event reporting in oncology. *Journal of Clinical Oncology*.

[46] Mozzicato P. (2009). Pharmaceutical medicine. *MedDRA*.

[47] Ma H., Hu Y., Zou Z., Feng M., Ye X., Li X. (2016). Antihyperglycemia and antihyperlipidemia effect of protoberberine alkaloids from rhizoma Coptidis in HepG2 cell and diabetic KK-ay mice. *Drug Development Research*.

[48] Kou S., Han B., Wang Y. (2016). Synergetic cholesterol-lowering effects of main alkaloids from Rhizoma Coptidis in HepG2 cells and hypercholesterolemia hamsters. *Life Sciences*.

[49] He K., Kou S., Zou Z. (2016). Hypolipidemic effects of alkaloids from rhizoma Coptidis in diet-induced hyperlipidemic hamsters. *Planta Medica*.

[50] Wang Y., Yi X., Ghanam K., Zhang S., Zhao T., Zhu X. (2014). Berberine decreases cholesterol levels in rats through multiple mechanisms, including inhibition of cholesterol absorption. *Metabolism*.

[51] Liu X., Yang Z., Li H. (2020). Chrysophanol alleviates metabolic syndrome by activating the SIRT6/AMPK signaling pathway in Brown adipocytes. *Oxidative Medicine and Cellular Longevity*.

[52] Wood L., Egger M., Gluud L. L. (2008). Empirical evidence of bias in treatment effect estimates in controlled trials with different interventions and outcomes: meta-epidemiological study. *BMJ*.

[53] Alshurafa M., Briel M., Akl E. A. (2012). Inconsistent definitions for intention-to-treat in relation to missing outcome data: systematic review of the methods literature. *PLoS One*.

[54] Moher D., Schulz K. F., Altman D. G. (2001). The CONSORT statement: revised recommendations for improving the quality of reports of parallel-group randomised trials. *The Lancet*.

[55] Hróbjartsson A., Gøtzsche P. C. (2010). Placebo interventions for all clinical conditions. *Cochrane Database of Systematic Reviews*.

[56] Zhang X., Tian R., Zhao C., Tang X., Lu A., Bian Z. (2019). Placebo design in WHO-registered trials of Chinese herbal medicine need improvements. *BMC Complementary and Alternative Medicine*.

[57] Hall K. D., Bemis T., Brychta R. (2015). Calorie for calorie, dietary fat restriction results in more body fat loss than carbohydrate restriction in people with obesity. *Cell Metabolism*.

[58] Baillot A., Romain A. J., Boisvert-Vigneault K. (2015). Effects of lifestyle interventions that include a physical activity component in class II and III obese individuals: a systematic review and meta-analysis. *PLoS One*.

[59] Zhou K., Zhang J., Xu L., Lim C. E. D. (2021). Chinese herbal medicine for subfertile women with polycystic ovarian syndrome. *Cochrane Database of Systematic Reviews*.

