# Supplementary material for: The Effects and Safety of Chinese Herbal Medicine on Blood Lipid Profiles in Placebo-Controlled Weight-Loss Trials: A Systematic Review and Meta-Analysis
Source: Evid Based Complement Alternat Med. 2022 Jan 17;2022:1368576. doi: 10.1155/2022/1368576 (PMC8786479; doi:10.1155/2022/1368576)
Supplement: Supplementary Materials — Contents of the supplementary file include the following tables and figures. Table S1: search strategy example used to retrieve citations from OVID MEDLINE database. Table S2: details of CHM and placebo intervention regimes. Table S3: comparison of changes in blood lipid profiles within and between CHM and placebo groups. Table S4: sensitivity analysis of 15 included study showing recalculation of effect estimates with correlation coefficients of 0.25, 0.50 (main meta-analysis), and 0.75. Figure S1: risk of bias summary of individual studies included in the meta-analysis based on Cochrane's Risk of Bias 2 Tool. Figure S2: leave-one-out analysis to detect various outliers for total cholesterol, triglyceride, LDL cholesterol, and HDL cholesterol outcomes. Figure S3: forest plots after significant outlier removed for total cholesterol, LDL cholesterol, and HDL cholesterol outcomes. Figure S4: funnel plots with trim-and-fill analysis for change from baseline total cholesterol, triglyceride, LDL cholesterol, and HDL cholesterol outcomes. [file 1368576.f1.docx]

**The effects and safety of Chinese herbal medicine on blood lipid profiles in placebo-controlled weight-loss trials: A systematic review and meta-analysis**

## **Supplementary appendix**

**Table S1**. Search strategy used to retrieve citations from OVID MEDLINE database.

| **14 Apr 2021 (177 results)** | | |
| --- | --- | --- |
| **Ovid Medline (1946 − 2020)** | | |
| #1 | 247,078 | (overweight or obes* or weight*).ti. |
| #2 | 190,312 | ("Traditional Chinese Medicine" or "Chinese Herbal Medicine" or "TCM" or "phytotherapy" or "Kampo" or herb*).mp. |
| #3 | 206,941 | (pill or powder or capsule or tablet or granule or decoction).mp. |
| #4 | 385,990 | 2 or 3 |
| #5 | 1,285,789 | (randomised controlled trial or controlled clinical trial).pt. or random*.ab. or clinical trials.sh. or trial.ti. |
| #6 | 3,381,536 | ((human or adult) not (infant* or child* or adolescent or animal or rodent* or rat* or mice)).sh. |
| #7 | 171 | 1 and 4 and 5 and 6 |

**Table S2**. Details of intervention ingredients and regime

| **Author** | **Year** | **Formula name** | **Ingredients** | **Regimen** |
| --- | --- | --- | --- | --- |
| Cheon | 2020 | Euiiyin-tang | Ephedra sinica Stapf (1.33 g), Angelica gigantis Radix (1.33 g), Atractylodis rhizoma Alba (1.33 g), Coicis Semen (3.33 g), Cinnamomi cortex (1.00 g), Paeonia lactiflora (1.00 g), Glycyrrhiza uralensis (0.67 g) | 1 dose (3g), tid |
|  |  | Placebo | Corn starch 1.51g, lactose hydrate 1.14g, citric acid hydrate 0.02g, hypromellose 0.21g, Croscarmellose sodium 0.06g, caramel color 0.06g, Ssanghwaflavor. |  |
| Cho (b) | 2013 | THI | Scutellariae Radix (~1.14g), Platycodi Radix (~1.14g), Oligosaccharide (3.18g), Berry flavor (0.05g) | 1 pack (50mL), tid |
|  |  | Placebo | Oligosaccharide, Berry flavor |  |
| Cho (a) | 2017 | YY-312 | Imperata cylindrical (~900 mg), Citrus unshiu (~360 mg), Evodia officinalis (~540 mg), Cyclodextrin (600 mg) | 3 tab (400mg each), bid |
|  |  | Placebo | Not reported. |  |
| Chong | 2014 | IQP-GC-101 | Garcinia cambogia (650 mg), Camellia sinensis (100mg), Coffea arabica (75 mg), Lagerstroemia speciosa (25 mg), 5.10g/d | 3 tab (850mg), bid |
|  |  | Placebo | Microcrystalline cellulose, dicalcium phosphate |  |
| Chung | 2016 | QXD | Scutellariae Radix (0.28 g), Coptidis Rhizoma (0.28 g), Phellodendri Cortex (0.28 g), Gardeniae Fructus (0.28 g), Rhei Rhizoma (0.07 g) | 3 cap (300mg each) (daily dose) |
|  |  | Placebo | Phenylthiocarbomide, Squid ink, Herbal flavor, Starch |  |
| Hioki | 2004 | Bofu-tsusho-san | Scutellariae Radix (44.4 g), Glycyrrhizae Radix (44.4 g), Platycodi Radix (44.4 g), Gypsum Fibrosum (44.4 g), Atractylodis Rhizoma (44.4 g), Rhei Rhizoma (33.3 g), Schizonepetae Spica (26.7 g), Gardeniae Fructus (26.7 g), Paeoniae Radix (26.7 g), Cnidium Rhizoma (26.7 g), Angelicae Radix (26.7 g), Menthae Herba (26.7 g), Ledebouriellae Radix (26.7 g), Ephedrae Herba (26.7 g), Forsythiae Fructus (26.7 g), Zingiberis Rhizoma (6.7 g), Talcum (66.7 g), Natrium Sulphuricum (15.6 g) per 100g dry extract, and active drug contains ephedrine (24mg/d) and caffeine (280mg/d) | 1 dose, tid |
|  |  | Placebo | NR |  |
| Lenon | 2012 | RCM-104 | Camellia sinensis (~2.4g), Cassia obtusifolia (~2.4g), Sophora japonica (~1.2g) | 4 cap (500mg each), tid |
|  |  | Placebo | Herbal starch |  |
| Park | 2013 | TJ001 | Semen Coicis (3.75 g), Semen Castaneae (3.75 g), Semen Raphani (2.5 g), Schisandrae Fructus (1.25 g), Liriopis tuber (1.25 g), Herba Ephedrae (1.25 g), Radix platycodi (1.25 g), Acori Tatarinowii Rhizoma (1.25 g) | 1 dose (7g), tid |
|  |  | Placebo | NR |  |
| Sheng | 2017 | Jianpi Shugan Jiangzhifang Fang | 苍术 Atractylodis Rhizoma, 柴胡 Bupleuri Radix, 半夏 Pinelliae Rhizoma, 香附 Cyperi Rhizoma，茯苓 Poria, 泽泻 Alismatis Rhizoma, 决明子 Cassiae Semen, 荷叶 Nelumbinis Folium [dose unspecified] | 1 dose, bid |
|  |  | Placebo | NR |  |
| Sun | 2017 | Yiqi Huatan Huoxue Zhongyao Fufang | 黄芪 Astragali Radix (20g), 丹参 Salviae Miltiorrhizae Radix et Rhizoma (15g), 苍术 Atractylodis Rhizoma (15g), 山楂 Crataegi Fructus (15g), 茯苓 Poria (15g), 续断 Dipsaci Radix (15g), 狗脊 Cibotii Rhizoma (10g), 茵陈 Artemisiae Scopariae Herba (15g), 黄连 Coptidis Rhizoma (5g), 陈皮 Citri Reticulatae Pericarpium (5g), 枳壳 Aurantii Fructus (10g), 薄荷 Menthae Haplocalycis Herba (10g) | 1/2 pack (100mL), bid |
|  |  | 5% Intervention | 5% original dose + 大米 (rice) |  |
| Tang | 2007 | Soufeng Shunqi Wan | 大黄 Rhei Radix et Rhizoma, 火麻仁 Cannabis Fructus, 郁李仁 Pruni Semen, 枳壳 Aurantii Fructus, 山茱萸 Corni Fructus, 车前子 Plantaginis Semen, 槟榔 Arecae Semen, 山药 Dioscoreae Rhizoma, 怀牛膝 Achyranthis Bidentatae Radix, 胆南星 Arisaema Cum Bile, 山楂 Crataegi Fructus [dose unspecified] | 1 dose (9g), tid |
|  |  | Placebo | 玉米面 corn flour |  |
| Wang | 2016 | Yiqi Huaju Fang | 黄芪 Astragali Radix (10g), 黄连 Coptidis Rhizoma (3g), 蒲黄 Typhae Pollen (10g), 茵陈 Artemisiae Scopariae Herba (10g), 泽泻 Alismatis Rhizoma (10g) | 1 sachet (17.5g), bid |
|  |  | 5% Intervention | 5% original dose |  |
| Wang | 2007 | Jianfei Heji | 山楂 Crataegi Fructus (~4.5g), 荷叶 Nelumbinis Folium (~9g), 泽泻 Alismatis Rhizoma (~4.5g) | 1 sachet (6g), tid |
|  |  | Placebo | 糊精 |  |
| Xu | 2008 | Hefeiqi Jiaonang | 北杏仁 Armeniacae Semen Amarum, 苍术 Atractylodis Rhizoma, 薏苡仁 Coicis Semen, 桃仁 Persicae Semen, 海藻 Sargassum [dose and other ingredients unspecified] | 2 cap, tid |
|  |  | Placebo | NR |  |
| Zhou | 2014 | XJXGF | Rhubarb, Coptis, Semen cassia and Citrus aurantium [dose and other ingredients unspecified] | 170mL, bid |
|  |  | 10% Intervention | 10% original dose |  |

NR, not reported; bid, twice daily; cap, capsule; qd, once daily; tab, tablet; tid, three times daily.

**Table S3.** Comparison of changes in blood lipid profiles within CHM and placebo groups as well as between CHM and placebo groups

| **Blood lipids (mmol/L)** | **No. of trials** | **No. of participants in CHM : placebo groups** | **Changes within CHM groups from baseline, MD [95% CI] I^2^** | **Changes within placebo groups from baseline, MD [95% CI] I^2^** | **Changes between CHM and placebo groups, MD [95% CI] I^2^** |
| --- | --- | --- | --- | --- | --- |
| Total cholesterol | 13 | 621: 602 | −0.34 [−0.64, −0.04]* 87% | −0.10 [−0.34, 0.15] 80% | −0.18 [−0.50, 0.14] 92% |
| Triglycerides | 15 | 776: 757 | −0.4 [−0.63, −0.17]* 87% | −0.15 [−0.29, −0.02]* 53% | −0.21 [−0.41, −0.02]* 81% |
| LDL-cholesterol | 13 | 598: 582 | −0.29 [−0.51, −0.08]* 81% | −0.09 [−0.29, 0.11] 76% | −0.09 [−0.24, 0.05] 67% |
| HDL-cholesterol | 15 | 776: 757 | 0.16 [0.04, 0.28]* 95% | 0.02 [−0.01, 0.05] 0% | 0.16 [0.04, 0.27]* 94% |

Note: * denotes significant p-value of <0.05. CHM, Chinese herbal medicine; CI, confidence intervals; HDL, high-density-lipoprotein; I^2^, test for heterogeneity in percentage; LDL, low-density lipoprotein; MD, mean difference. Data were pooled using the inverse variance method fitted in a random-effects model expressed as mean difference (MD) and 95% confidence intervals (CIs), and between-study variance was estimated with DerSimonian and Laird; two-tailed significance was set at a value of p < 0.05; number of participants.

**Table S4**. Sensitivity analysis of 15 included studies showing recalculation of effect estimate adopting correlation coefficients of 0.25, 0.50 (main meta-analysis) and 0.75.

| **Blood lipids (mmol/L)** | **No. of trials (participants) for changes between CHM and placebo** | **Correlation coefficient of 0.25**  **Changes between CHM and Placebo groups, MD [95% CI] *I*^2^** | **Correlation coefficient of 0.5**  **Changes between CHM and Placebo groups, MD [95% CI] *I*^2^** | **Correlation coefficient of 0.75**  **Changes between CHM and Placebo groups, MD [95% CI] *I*^2^** |
| --- | --- | --- | --- | --- |
| **Total cholesterol** | 13 (1223) | -0.17 [-0.46, 0.12] 88% | -0.18 [-0.50, 0.14] 92% | -0.19 [-0.55, 0.18] 95% |
| **Triglycerides** | 15 (1533) | -0.21 [-0.40, −0.01]* 77% | -0.21 [-0.41, −0.02]* 81% | -0.22 [-0.42, −0.02]* 87% |
| **LDL-cholesterol** | 13 (1180) | -0.07 [-0.20, 0.07] 54% | -0.09 [-0.24, 0.05] 67% | -0.11 [-0.27, 0.05] 80% |
| **HDL-cholesterol** | 15 (1533) | 0.15 [0.05, 0.26]* 91% | 0.16 [0.04, 0.27]* 94% | 0.16 [0.03, 0.29]* 97% |

* CHM, Chinese herbal medicine; CI, confidence intervals; HDL, high-density-lipoprotein; *I*^2^, test for heterogeneity in percentage; LDL, low-density-lipoprotein; MD, mean difference.


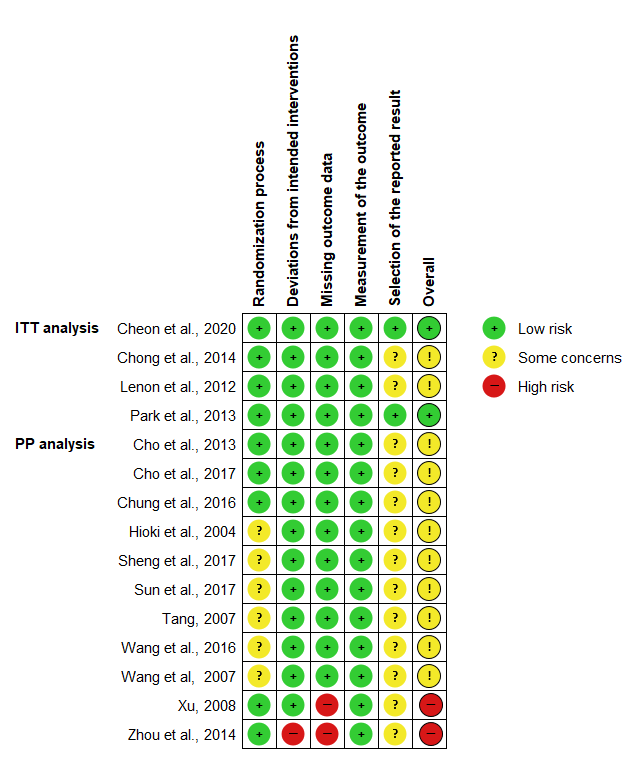


**Figure S1.** Risk of bias assessment summary of individual studies included in meta-analysis based on five domains according to Cochrane’s RoB 2 tool. ITT, intention-to-treat analysis (includes modified ITT); PP, per-protocol analysis.


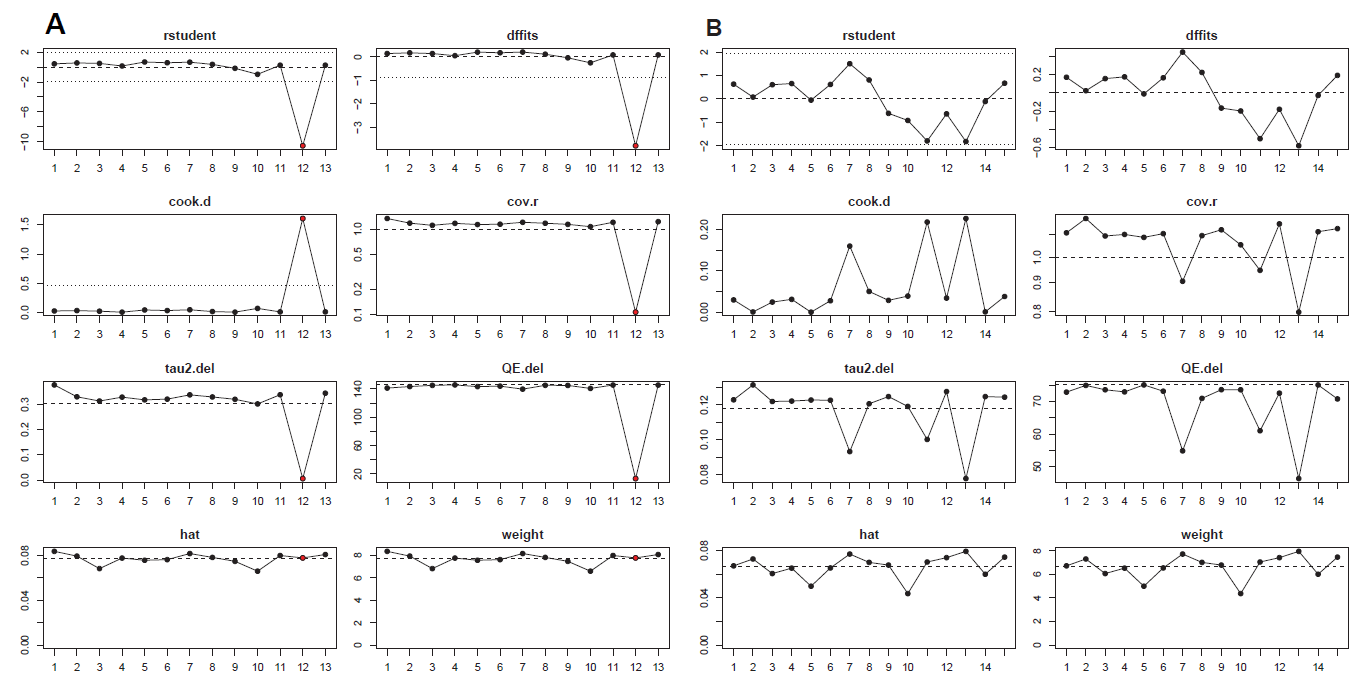


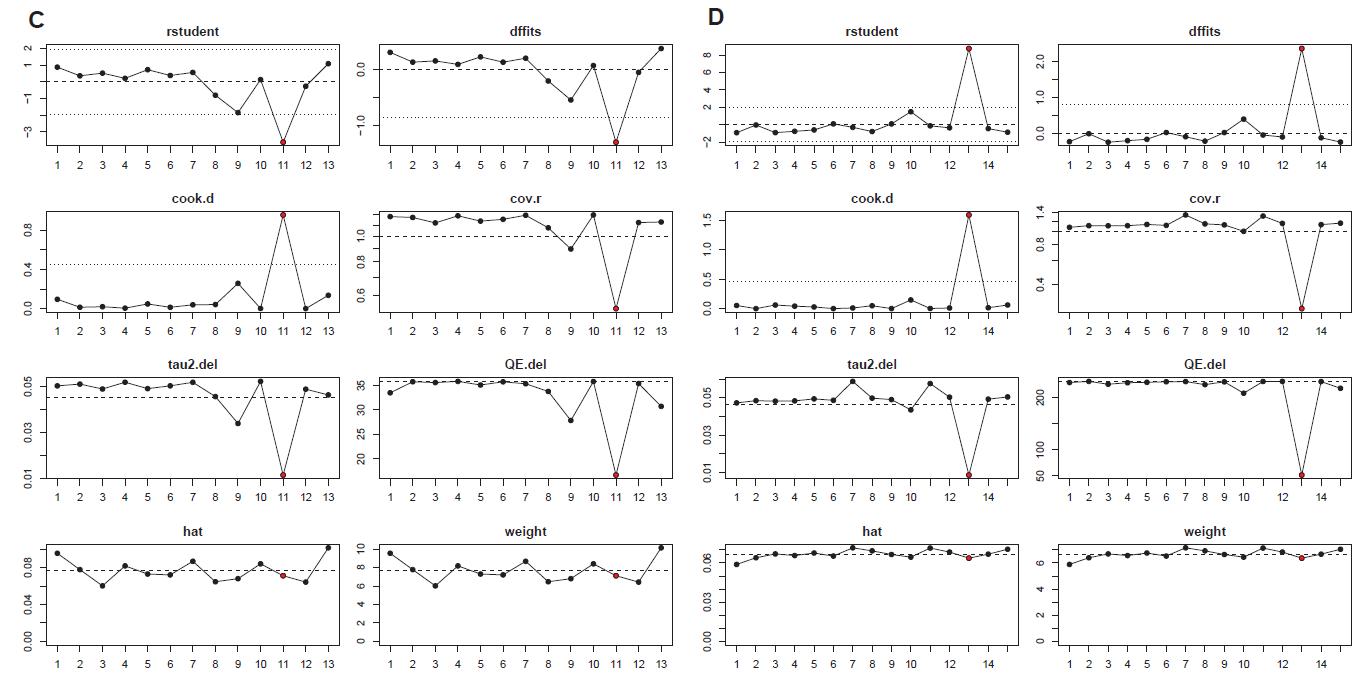


**Figure S2**. Leave-one-out diagnostics was performed to detect various outliers for (**A**) total cholesterol, (**B**) triglycerides, (**C**) LDL-cholesterol, (**D**) HDL-cholesterol. Red dots indicate significant outliers and were removed from meta-analysis for recalculation of effect estimates as part of sensitivity analysis. rstudent, externally standardized residuals; dffits, DFFITS values; cook.d, Cook’s distances; cov.r, covariance ratios, tau2.del, estimates of τ2 when each study is removed in turn; QE.del, the test statistics for (residual) heterogeneity when each study is removed in turn; hat, the diagonal elements of the hat matrix; weight, weights (in %) given to the obser3Aved outcomes during the model fitting.

**A**


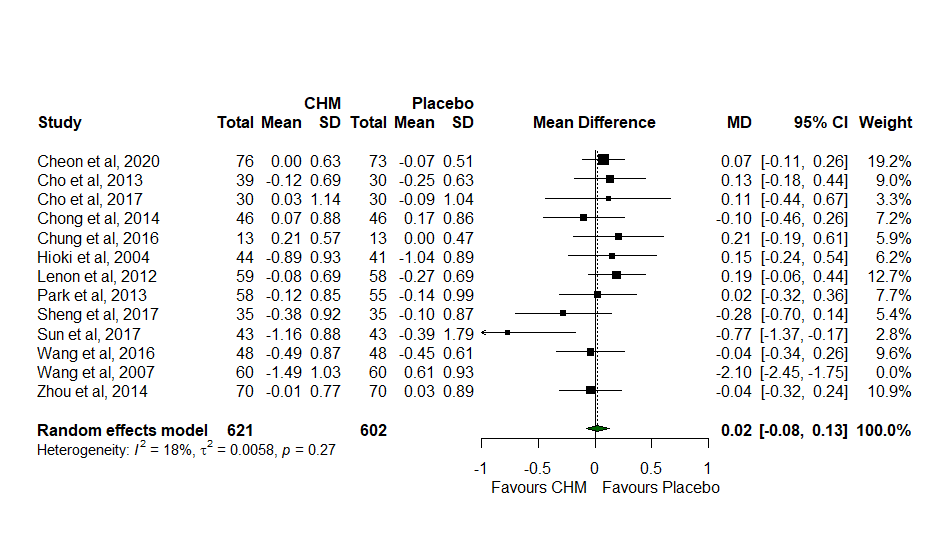


**B**


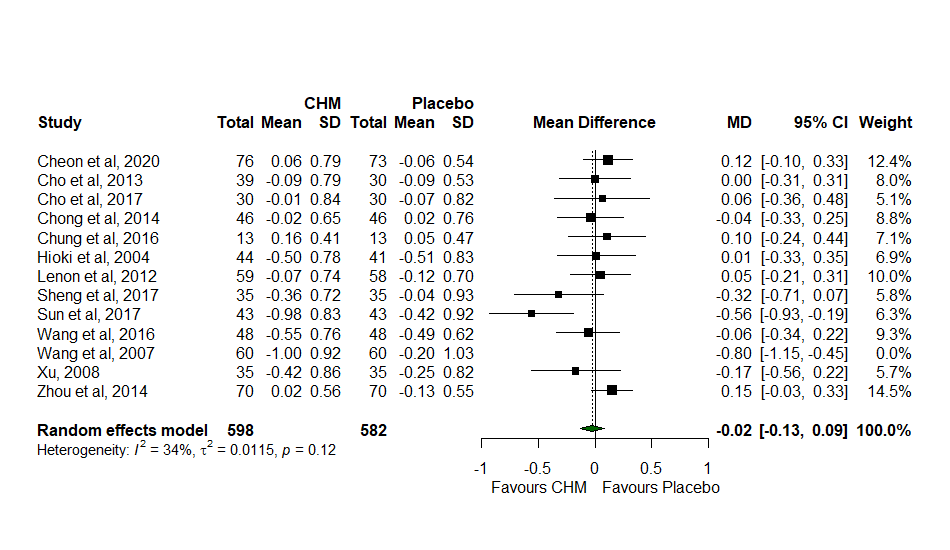


**C**


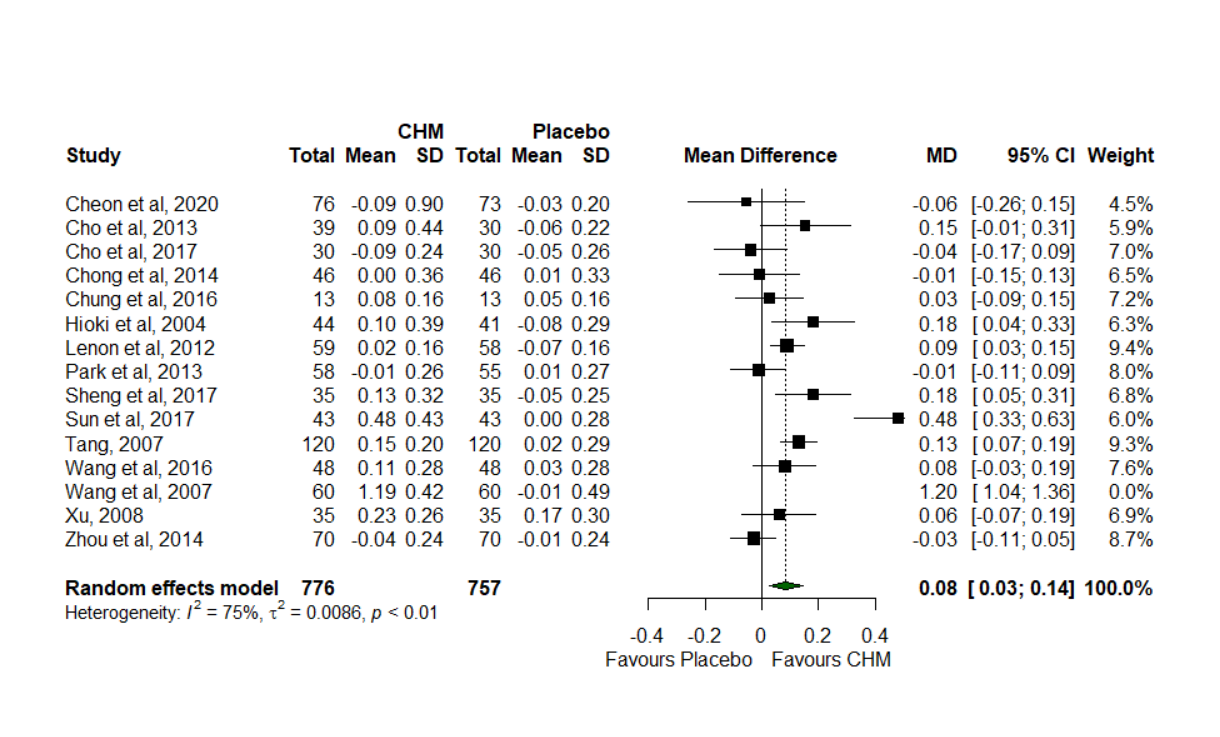


**Figure S3**. Forest plots after significant outlier removed for (**A**) total cholesterol, (**B**) LDL-cholesterol, and (**C**) HDL-cholesterol. Subgroup analysis of included trials investigating the effect of Chinese herbal medicine (CHM) and placebo on change in HDL-cholesterol. Data were pooled using the inverse variance method fitted in a random-effects model expressed as mean difference (MD) and 95% confidence intervals (CIs), and between-study variance was estimated with DerSimonian and Laird; two-tailed significance was set at a value of p < 0.05; n, number of participants.


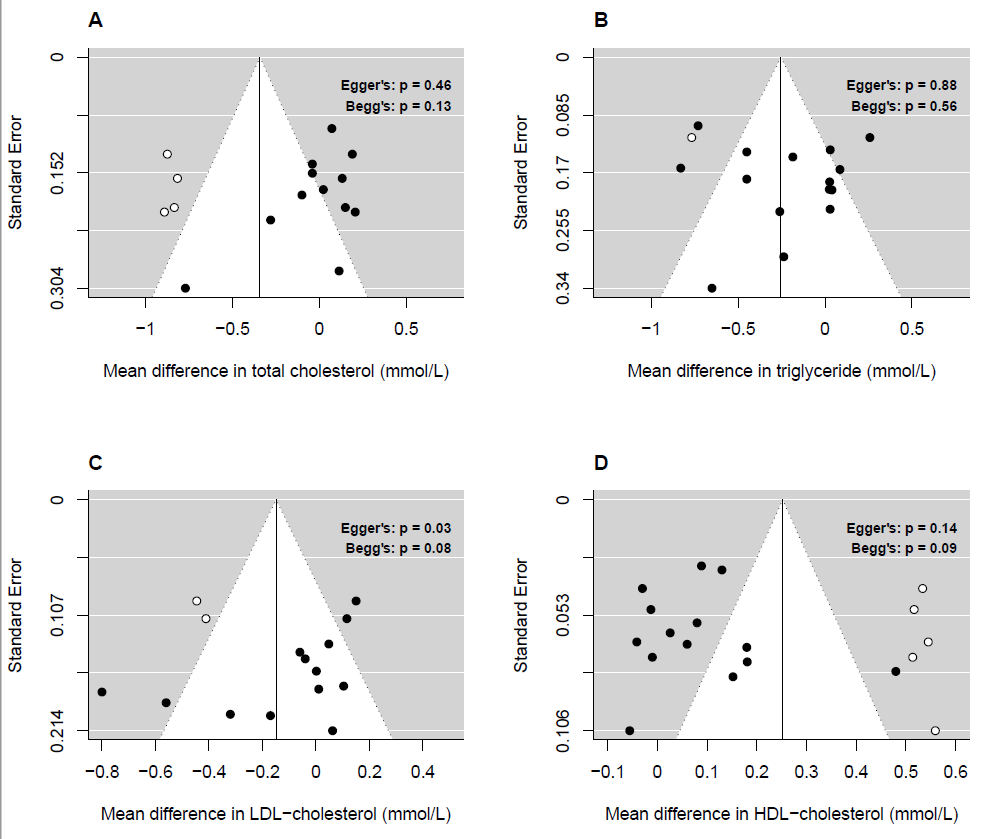


**Figure S4.** Funnel plots with trim-and-fill-analysis for change from baseline total cholesterol (**A**), triglyceride (**B**), LDL-cholesterol (**C**), and HDL-cholesterol (**D**) outcomes. The pooled effect estimate (mean difference) is represented by a solid line, framed by the dotted pseudo-95% confidence limits. Egger’s regression test was performed using standard error prediction in a random-effects model (DerSimonian and Laird estimator). Significance was set at *p* < 0.05.
